# The association between heart rhythm complexity and the severity of abdominal aorta calcification in peritoneal dialysis patients

**DOI:** 10.1038/s41598-018-33789-x

**Published:** 2018-10-23

**Authors:** Cheng-Hsuan Tsai, Chen Lin, Yi-Heng Ho, Men-Tzung Lo, Li-Yu Daisy Liu, Chih-Ting Lin, Jenq-Wen Huang, Chung-Kang Peng, Yen-Hung Lin

**Affiliations:** 1 Department of Internal Medicine, National Taiwan University Hospital and National Taiwan University College of Medicine, Taipei, Taiwan; 2 Biomedical Engineering, National Taiwan University Hospital and National Taiwan University College of Medicine, Taipei, Taiwan; 3 Center for Dynamical Biomarkers and Translational Medicine, National Central University, Chungli, Taiwan; 4 Department of Electrical Engineering, National Central University, Chungli, Taiwan; 5 Graduate Institute of Biomedical Electronics and Bioinformatics, National Taiwan University, Taipei, Taiwan; 6 Department of Agronomy, Biometry Division, National Taiwan University, Taipei, Taiwan; 7 Division of Interdisciplinary Medicine and Biotechnology, Beth Israel Deaconess Medical Center/Harvard Medical School, Boston, Massachusetts, USA

## Abstract

Abdominal aorta calcification (AAC) has been associated with clinical outcomes in peritoneal dialysis (PD) patients. Heart rhythm complexity analysis has been shown to be a promising tool to predict outcomes in patients with cardiovascular disease. In this study, we aimed to analyze the association between heart rhythm complexity and AAC in PD patients. We prospectively analyzed 133 PD patients. Heart rhythm complexity including detrended fluctuation analysis and multiscale entropy was performed. In linear analysis, the patients in the higher AAC group (AAC ≥15%) had a significantly lower standard deviation of normal RR intervals, very low frequency, low frequency, high frequency and low/high frequency ratio. In non-linear analysis, DFAα1, slope 1–5, scale 5 and area 6–20 were significantly lower in the patients with higher AAC. Receiver operating characteristic curve analysis showed that DFAα1 had the greatest discriminatory power to differentiate these two groups. Multivariate logistic regression analysis showed that DFAα1 and HbA1c were significantly associated with higher AAC ratio. Adding DFAα1 significantly improved the discriminatory power of the linear parameters in both net reclassification improvement and integrated discrimination improvement models. In conclusion, DFAα1 is highly associated with AAC and a potential cardiovascular marker in PD patients.

## Introduction

Cardiovascular disease (CVD) is the leading cause of morbidity and mortality in end-stage renal disease (ESRD) patients^1^, accounting for about 40% of deaths among these patients during the first 3 years of dialysis^2^. Because of the high cardiovascular mortality rate in dialysis patients (almost 5–30 times greater than in the general population)^3^, the burden of CVD on patients starting dialysis and its impact on the survival of dialysis patients has recently received increasing attention^2,3^.

Possible explanations for the high rates of CVD mortality and mobility in ESRD patients include atherosclerosis-related vascular complications and autonomic nervous system dysfunction^4^. Abdominal aorta calcification (AAC) has been reported to predict CVD events and mortality in ESRD patients, including those undergoing peritoneal dialysis (PD)^5–7^. The abdominal aorta calcification can be measured via X ray with grading systems^8^ or computed tomography (CT) with direct measurement of the percentage of AAC (%AAC)^9^. The %AAC has been shown to be independently associated with mortality and hospitalization in PD patients^7^. The cutoff value of 15% AAC in CT has been reported that predicting clinical outcomes in PD patients^7^. However, the clinical use of %AAC measurement is limited by radiation exposure and medical cost.

Analysis of beat-to beat variation of heart rate, also known as heart rate variability (HRV), is commonly used in cardiovascular researches as a simple and noninvasive approach^10^. HRV has also been commonly used to predict CVD outcomes^11^. Newer biological signal analysis methods based on nonlinear signal modeling and complexity evaluation including detrended fractal analysis (DFA) and multiscale entropy (MSE) have been developed in recent years^12^. Compared to traditional HRV parameters, nonlinear heart rhythm complexity analysis has a better prognostic power in patients with CVD. In addition, both DFA and MSE have been shown to be useful in predicting survival of heart failure patients^13,14^.

To the best of our knowledge, no previous study has investigated the association between heart rhythm complexity and AAC. Therefore, the aim of this study was to analyze the association between heart rhythm complexity and the severity of AAC in PD patients.

## Results

### Patients

A total of 133 PD patients (61 men) were enrolled in this study, including 59 (26 men) with AAC ≥15% (higher AAC group) and 74 (35 men) with AAC <15% (lower AAC group). The clinical data are shown in Table 1. The AAC ratio of the whole population, AAC ≥15% group and AAC <15% were 10.38 (0.53–30.70), 33.87 (27.25–46.39) and 1.32 (0.00–8.74), respectively. Patients in higher AAC group were significantly older and had higher incidences of diabetes mellitus (DM), HbA1c, fasting serum glucose, C-reactive protein (CRP), and lower serum creatinine and left ventricular ejection fraction (LVEF). Other clinical parameters including peritoneal dialysis efficiency (PD KT/V), percentage of beta-blocker and calcium channel blocker usage were comparable in both groups (Table 1).

**Table 1:** Clinical data of the patients.

	AAC <15%	AAC ≥15%	*p* value
	(N = 74)	(N = 59)	
Age(Years)	52.59 (43.44~59.47)	58.63 (51.27~64.84)	0.001
Male, n(%)	35 (47%)	26 (44%)	0.710
DM, n(%)	6 (8%)	22 (37%)	<0.001
HTN, n(%)	61 (82%)	52 (88%)	0.361
**Medication**			
ACEI or ARB	33 (45%)	32 (54%)	0.269
Beta-blocker	41 (55%)	36 (61%)	0.515
CCB	45 (61%)	44 (75%)	0.094
Statin	23 (31%)	23 (39%)	0.341
Glucose AC, mg/dL	91.00 (85.75~104.25)	106.00 (92.00~140.00)	<0.001
HbA1c, %	5.30 (5.00~5.65)	6.00 (5.30~7.00)	<0.001
Creatinine, mg/dL	11.75 (9.58~13.58)	10.40 (8.90~12.70)	0.037
PD KT/V	1.87 (1.67~2.05)	1.92 (1.67~2.17)	0.203
TG, mg/dL	151.50 (86.75~227.00)	167.00 (101.00~240.00)	0.377
T-Chol, mg/dL	193.00 (166.00~233.00)	181.00 (149.00~219.00)	0.118
LDL, mg/dL	90.50 (62.75~111.75)	77.00 (61.00~107.00)	0.400
HDL, mg/dL	39.00 (33.00~50.25)	36.00 (31.00~43.00)	0.106
Na, mmol/L	136.00 (133.00~138.00)	136.00 (132.00~138.00)	0.677
K, mmol/L	3.90 (3.40~4.30)	3.80 (3.10~4.20)	0.187
Ca, mg/dL	9.78 (9.00~10.29)	9.52 (8.88~9.92)	0.155
P, mg/dL	5.20 (4.80~6.30)	5.20 (4.40~6.10)	0.612
CRP, mg/dL	0.24 (0.10~0.79)	0.52 (0.17~1.54)	0.023
LVEF, %	70.53 (63.80~75.86)	65.98 (57.71~73.54)	0.037
AAC, %	1.32 (0.00~8.74)	33.87(27.25~46.39)	<0.001

Data were presented as median (25th~75th percentile) or number (percentage). AAC = abdominal aorta calcification; DM = diabetes mellitus; HTN = hypertension; ACE-I = angiotensin converting enzyme inhibitor; ARB = angiotensin receptor blocker; CCB = calcium channel blocker; PD = peritoneal dialysis; TG = triglycerides; T-Chol = total cholesterol; LDL = Low-density lipoprotein; HDL = high-density lipoprotein; CRP = C-reactive protein; LVEF = left ventricular ejection fraction.

### Holter data

In linear analysis, the patients in the higher AAC group had a significantly lower standard deviation of normal RR intervals (SDRR), very low frequency (VLF), low frequency (LF), high frequency (HF) and low frequency to high frequency ratio (LH/HF ratio) than those in the lower AAC group. In non-linear analysis, DFAα1 was significantly lower in the higher AAC group. The value of DFAα2 was comparable between the two groups. In MSE analysis, the patients in the higher AAC group had significantly lower slope 1–5, scale 5, and area 6–20 than the patients in the lower AAC group (Table 2).

**Table 2:** Holter parameter of the patients with different AAC ratio.

	AAC <15% (N = 74)	AAC ≥15% (N = 59)	*p* value
**Time Domain Analysis**			
Mean RR, ms	761.29 (660.07~843.09)	780.67 (713.31~889.30)	0.084
SDRR, ms	46.11 (34.28~58.12)	33.67(23.31~46.99)	0.001
pNN50, %	0.33 (0.08~1.77)	0.48 (0.05~2.20)	0.515
pNN20, %	5.85 (2.15~17.06)	5.23 (1.34~16.44)	0.339
**Frequency Domain Analysis**			
VLF	789.84 (477.23~1459.70)	315.90 (154.40~678.98)	<0.001
LF	152.21 (71.19~295.19)	49.18 (17.30~147.01)	<0.001
HF	49.59 (19.05~118.55)	29.56 (11.24~68.32)	0.038
LF/HF ratio	2.60 (1.57~4.38)	1.33 (0.91~2.62)	<0.001
**Detrended fluctuation analysis**			
DFAα1	1.26 (1.13~1.43)	1.00 (0.85~1.15)	<0.001
DFAα2	1.24 (1.17~1.31)	1.22 (1.13~1.30)	0.853
**Multiscale entrophy**			
Slope 1–5	0.063 (0.0177~0.1011)	0.023 (−0.017~0.058)	<0.001
Scale 5	1.044 (0.898~1.197)	0.879 (0.722~1.069)	0.002
Area 1–5	4.494 (3.892~5.359)	4.22 (3.26~5.17)	0.122
Area 6–20	18.50 (16.21~21.59)	16.14 (13.79~19.26)	0.001

Values are median (25th~75th percentile). SDNN = standard deviation of normal RR intervals; pNN20 = percentage of the absolute change in consecutive normal RR interval exceeds 20 ms; pNN50 = percentage of the absolute change in consecutive normal RR interval exceeds 50 ms; VLF = very low frequency; LF: low frequency; HF = high frequency; DFA = detrended fluctuation analyses.

### Differentiation between the higher and lower AAC groups

**Figure 1:**
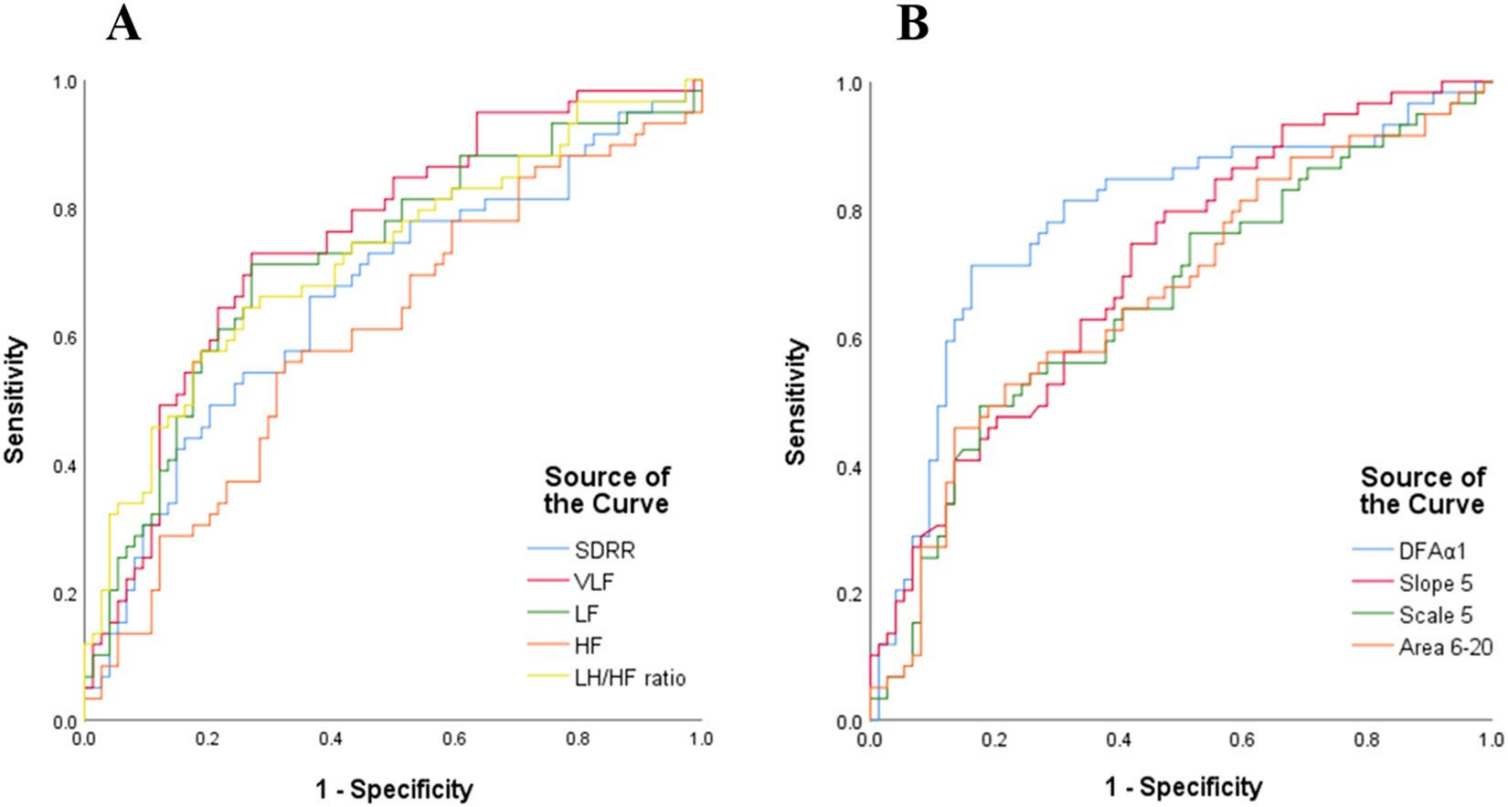
(**A**, **B**) Analysis of the discrimination power of linear and non-linear parameters to discriminate patients with higher AAC (AAC ≥15) by receiver operating characteristic curve analysis (**A**) The areas under the curve of SDRR, VLF, LF, HF, LF/HF ratio were 0.663, 0.752, 0.721, 0.605 and 0.723 respectively. (**B**) The areas under the curve of DFAα1, slope 5, scale 5 and area 6–20 were 0.781, 0.707, 0.657 and 0.667, respectively.

Receiver operating characteristic (ROC) curve analysis showed that DFAα1 had the greatest discriminatory power to differentiate the two groups compared to other linear, non-linear and clinical parameters (Fig. 1).

The areas under the curves (AUC) of HRV parameters including SDRR, the percentage of absolute differences in normal RR intervals greater than 50 ms (pNN50), pNN20, VLF, LF, HF, LF/HF ratio DFAα1, DFAα2, slope 1–5, scale 5, area 1–5, and area 6–20 were 0.663, 0.457, 0.548, 0.752, 0.721, 0.605, 0.723, 0.781, 0.509, 0.707, 0.657, 0.578 and 0.667, respectively.

The AUC of clinical parameters including age, DM, fasting serum glucose, HbA1c, creatinine, CRP and LVEF were 0.641, 0.651, 0.683, 0.729, 0.580, 0.622 and 0.619, respectively.

### Logistic regression analysis to predict the higher AAC group

In univariate logistic regression analysis, age, DM, fasting serum glucose, HbA1c, creatinine, LVEF, SDRR, VLF, LF, LF/HF ratio, DFAα1, slope 1–5, scale 5, and area 6–20 were significantly associated with the presence of higher AAC. In multivariate logistic regression analysis, only DFAα1 (OR = 0.032, 95% CI 0.005 to 0.212, *p* < 0.001) and HbA1c (OR = 3.497, 95% CI 1.727 to 7.084, *p* = 0.001) remained in the model, and both were associated with higher AAC (Table 3).

**Table 3:** Univariate and multivariate logistic regression model to predict higher abdominal aorta calcification group.

Abnormal aorta calcification				
Univariate logistic regression			Multivariate logistic regression	
	β (95% C.I)	*p* value	OR (95% C.I)	p value
Age	1.058 (1.024~1.094)	0.001		
Sex	1.139 (0.573~2.265)	0.710		
DM	6.74 (2.51~18.09)	<0.001		
HTN	1.583 (0.588~4.263)	0.363		
Glucose, mg/dL	1.023 (1.009~1.038)	0.002		
HbA1c, %	4.374 (2.339~8.181)	<0.001	3.497 (1.727~7.084)	0.001
PD KT/V	1.479 (0.567~3.854)	0.424		
Creatinine, mg/dL	0.842 (0.734~0.966)	0.014		
Ca, mg/dL	0.782 (0.539~1.137)	0.782		
P, mg/dL	0.927 (0.698~1.233)	0.604		
TG, mg/dL	1.000 (0.998~1.003)	0.633		
T-Chol, mg/dL	0.995 (0.988~1.003)	0.224		
LDL, mg/dL	0.998 (0.989~1.007)	0.696		
HDL, mg/dL	0.974 (0.944~1.005)	0.099		
CRP, mg/dL	0.993 (0.842~1.171)	0.934		
LVEF, %	0.964 (0.933~0.996)	0.027		
Mean RR	1.002 (1.000~1.005)	0.092		
SDRR	0.976 (0.959~0.994)	0.009		
pNN50	1.003 (0.966~1.042)	0.860		
pNN20	0.991 (0.969~1.014)	0.445		
VLF	0.998 (0.998~0.999)	<0.001		
LF	0.998 (0.997~1.000)	0.046		
HF	1.000 (0.999~1.002)	0.739		
LF/HF ratio	0.828 (0.709~0.967)	0.017		
DFAα1	0.021 (0.004~0.109)	<0.001	0.032 (0.005~0.212)	<0.001
DFAα2	0.600 (0.059~6.119)	0.667		
Slope 1–5	<0.001 (<0.001~0.001)	<0.001		
Scale 5	0.203 (0.059~0.705)	0.012		
Area 1–5	0.844 (0.661~1.078)	0.175		
Area 6–20	0.875 (0.801~0.956)	0.003		

PD = peritoneal dialysis; TG = triglycerides; T-Chol = total cholesterol; LDL = Low-density lipoprotein; HDL = high-density lipoprotein; CRP = C-reactive protein; LVEF = left ventricular ejection fraction; SDNN = standard deviation of normal RR intervals; pNN20 = percentage of the absolute change in consecutive normal RR interval exceeds 20 ms; pNN50 = percentage of the absolute change in consecutive normal RR interval exceeds 50 ms; VLF = very low frequency; LF = low frequency; HF = high frequency; DFA = detrended fluctuation analyses.

### Correlations of HRV parameters and percentage of AAC

In univariate linear regression analysis, age, DM, fasting serum glucose, HbA1c, creatinine, LVEF, SDRR, VLF, LF/HF ratio, DFAα1, slope 1–5, scale 5, and area 6–20 were significantly associated with the percentage of ACC. In the multivariate linear regression model, DFAα1 (β:−31.189, 95% CI −44.829 to −17.550, *p *< 0.001), LF/HF ratio (β:1.111, 95% CI 0.161 to 2.060, *p* = 0.022), age (β:0.293, 95% CI 0.057 to 0.529, *p* = 0.015) and HbA1c (β:4.744, 95% CI 1.640 to 7.847, *p* = 0.003) were significantly associated with the percentage of AAC (Table 4).

**Table 4:** Univariate and multivariate linear regression to predict percentage (%) of abdominal aorta calcification.

Abnormal aorta calcification				
Univariate linear regression			Multivariate linear regression	
	β (95% C.I)	*p* value	β (95% C.I)	*p* value
Age	0.557 (0.335~0.778)	<0.001	0.293 (0.057~0.529)	0.015
Sex	0.424 (−5.650~6.498)	0.890		
DM	9.537 (2.298~16.776)	0.010		
HTN	4.854 (−3.572~13.280)	0.256		
Glucose, mg/dL	0.193 (0.111~0.276)	<0.001		
HbA1c, %	8.110 (5.047~11.173)	<0.001	4.744 (1.640~7.847)	0.003
PD KT/V	4.508 (−3.821~12.838)	0.286		
Creatinine, mg/dL	−1.484 (−2.585~−0.384)	0.009		
Ca, mg/dL	−2.264 (−5.460~0.933)	0.164		
P, mg/dL	−0.755 (−3.256~1.745)	0.551		
TG, mg/dL	−0.032 (−0.099~0.034)	0.336		
T-Chol, mg/dL	0.011 (−0.007~0.028)	0.240		
LDL, mg/dL	−0.001 (−0.08~0.077)	0.970		
HDL, mg/dL	−0.241 (−0.502~0.019)	0.069		
CRP, mg/dL	0.249 (−1.201~1.700)	0.734		
LVEF, %	−0.339 (−0.602~−0.077)	0.012		
Mean RR	0.011 (−0.011~0.033)	0.320		
SDRR	−0.153 (−0.291~−0.015)	0.030		
pNN50	0.119 (−0.219~0.458)	0.486		
pNN20	−0.039 (−0.231~0.154)	0.693		
VLF	−0.008 (−0.012~−0.004)	<0.001		
LF	−0.01 (−0.021~0.001)	0.081		
HF	0.006 (−0.010~0.022)	0.481		
LF/HF ratio	−0.982 (−1.758~−0.206)	0.013	1.111 (0.161~2.060)	0.022
DFAα1	−28.305 (−37.519~−19.091)	<0.001	−31.189 (−44.829~-17.550)	<0.001
DFAα2	−6.613 (−27.159~13.933)	0.525		
Slope 1–5	−84.854 (−128.136~−41.572)	<0.001		
Scale 5	−13.554 (−22.788~−4.32)	0.004		
Area 1–5	−1.816 (−3.852~0.220)	0.08		
Area 6–20	−1.116 (−1.777~−0.455)	0.001		

Adjusted R2:0.346. PD = peritoneal dialysis; TG = triglycerides; T-Chol = total cholesterol; LDL = Low-density lipoprotein; HDL = high-density lipoprotein; CRP = C-reactive protein; LVEF = left ventricular ejection fraction; SDNN = standard deviation of normal RR intervals; pNN20 = percentage of the absolute change in consecutive normal RR interval exceeds 20 ms; pNN50 = percentage of the absolute change in consecutive normal RR interval exceeds 50 ms; VLF = very low frequency; LF = low frequency; HF = high frequency; DFA = detrended fluctuation analyses.

### The advantage of adding DFA or MSE parameters to the linear parameters to discriminate the higher and lower AAC groups

DFAα1 and slope 1–5 significantly improved the discriminatory power of SDRR, VLF, LF, HF and LF/HF ratio in both net reclassification improvement (NRI) and integrated discrimination improvement (IDI) models. In addition, area 6–20 significantly improved the discriminatory power of SDRR, LF, HF and LF/HF ratio in the IDI model, and SDRR and HF in the NRI model. Scale 5 significantly improved the discriminatory power of SDRR, HF and LF/HF ratio in the IDI model and HF in NRI model (Table 5).

**Table 5:** AUC, NRI, and IDI models of linear parameters before and after adding DFAα1 and MSE parameters.

Parameters		AUC	R square	NRI	NRI *p*-value	IDI	IDI *p*-value
SDRR		0.663	0.055				
	+DFAα1	0.783	0.213	0.863	<0.001	0.165	<0.001
	+Slope1–5	0.728	0.157	0.491	0.004	0.102	<0.001
	+Area6–20	0.711	0.105	0.362	0.034	0.051	0.007
	+Scale 5	0.705	0.083	0.186	0.281	0.031	0.031
VLF		0.752	0.141				
	+DFAα1	0.795	0.233	0.477	0.005	0.082	0.001
	+Slope1–5	0.773	0.202	0.383	0.025	0.052	0.008
	+Area6–20	0.768	0.168	0.085	0.619	0.023	0.071
	+Scale 5	0.761	0.153	0.071	0.680	0.008	0.278
LF		0.721	0.034				
	+DFAα1	0.782	0.201	0.849	<0.001	0.173	<0.001
	+Slope1–5	0.711	0.140	0.410	0.016	0.098	<0.001
	+Area6–20	0.693	0.076	0.261	0.132	0.040	0.023
	+Scale 5	0.698	0.054	0.315	0.068	0.022	0.080
HF		0.605	0.001				
	+DFAα1	0.783	0.198	0.978	<0.001	0.213	<0.001
	+Slope1–5	0.714	0.151	0.599	<0.001	0.151	<0.001
	+Area6–20	0.673	0.087	0.538	0.001	0.087	<0.001
	+Scale 5	0.670	0.083	0.497	0.003	0.083	0.001
LF/HF ratio		0.722	0.050				
	+DFAα1	0.793	0.214	1.005	<0.001	0.167	<0.001
	+Slope1–5	0.708	0.140	0.403	0.019	0.075	0.002
	+Area6–20	0.709	0.090	0.234	0.177	0.038	0.027
	+Scale 5	0.719	0.082	0.234	0.177	0.034	0.033

SDRR = standard deviation of normal RR intervals; VLF = very low frequency; LF = low frequency; HF = high frequency; AUC = areas under the curve; NRI = net reclassification improvement; IDI = integrated discrimination improvement; MSE = multiscale entropy; DFA = detrended fluctuation analyses.

## Discussion

There were three major findings in this study. First, the PD patients with higher AAC had worse heart rhythm complexity. Second, in all linear and non-linear parameters, DFAα1 had the greatest single discriminatory power to detect PD patients with higher AAC. Third, non-linear parameters, especially DFAα1, significantly improved the discriminatory power of the linear parameters to differentiate PD patients with higher or lower AAC.

In daily practice, predicting the clinical outcomes of PD patients is a challenge. Atherosclerosis-related vascular calcification has been highly associated with morbidity and mortality in ESRD patients^5,6,15^. In the advanced stage of atherosclerosis such as atheroma formation, a partial or extensive calcium deposit is frequently observed^16^. Therefore, blood vessel calcification implies the presence atherosclerosis or subclinical CVD^17,18^. Several traditional risk factors for atherosclerosis such as dyslipidemia, hypertension, smoking, and age have also been associated with vascular calcification in ESRD patients^19,20^. In addition, uremia, mineral metabolism, chronic inflammation, fetuin-A and osteoprotegerin (OPG) have also been reported to contribute to vascular calcification^21,22^. Several studies have reported significant associations between qualitative or semiquantitative evaluations of arterial calcification and all-cause and cardiovascular mortality in hemodialysis patients^23–25^. In our study, HbA1c and age were significantly associated with %AAC in multivariate linear regression model. Age and HbA1c are known risk factors of vascular calcification^26,27^. In addition, HbA1c levels are associated with mortality in ESRD patients^28,29^. Even in PD patients without diabetes, higher HbA1c is still associated with higher cardiovascular events^30^.

The AAC severity measurements include X ray with Kauppila score^8^ and CT with direct measurement of %AAC^9^. Previous study supported that CT appeared to be more sensitive than plain X-rays at detecting peripheral and aortic vascular calcifications in hemodialysis patients^31^. Tsushima *et al*. developed a method to measure the percentage of calcified volume against whole vascular volume using CT^9,32^ and CT remains the reference standard in AAC evaluation^33^. AAC was reported to be an important predictor of vascular morbidity and mortality in the Framingham Heart Study^34^, and it has also been reported to be associated with clinical outcomes in ESRD patients^5–7^. The percentage of AAC has been shown to be independently associated with mortality and hospitalization in PD patients^7^. In addition, patients with AAC ≥15% had more cardiovascular events than those with AAC <15%^7^. However, despite the usefulness of %AAC by CT, the radiation exposure and cost were limited the use of this tool.

In contrast, electrocardiography (ECG) is an easy, low cost and radiation-free examination. In the present study, we found high correlations among the HRV parameters (especially DFAα1) and AAC. This indicates that ECG recording followed by HRV analysis using linear and non-linear parameters has the potential to be an alternative to AAC in clinical practice.

Previous studies have reported an association between the progression of coronary and carotid artery atherosclerosis and autonomic dysfunction^35,36^. Despite the reported association between autonomic dysfunction and atherosclerosis^37^, the mechanisms linking autonomic imbalance to atherosclerosis are still unclear. In addition to atherosclerosis-related autonomic dysfunction, uremic autonomic neuropathy in ESRD patients has frequently been associated with parasympathetic damage and sympathetic nerve overactivity^38^, both of which have been associated with worse clinical outcomes in ESRD patients^39^. In our previous study, PD patients had significantly lower values of several linear and nonlinear parameters than those with normal renal function, and this also supports the hypothesis of prominent autonomic dysfunction in ESRD patients^40^.

In the present study, DFAα1 had a better correlation with AAC than linear parameters, which implies that non-linear parameters provide more useful information. The non-linear analysis of HRV including MSE and DFA has been reported to be a better predictor of clinical outcomes than traditional linear analysis. MSE has been associated with the prognosis of heart failure^13^, outcome of acute stroke^41^, primary aldosteronism^42^, critical illnesses requiring extracorporeal life support^43^, and post-myocardial infarction heart function^44^. Furthermore, long-time scale parameters (area 6–20) in heart failure patients have been shown to have the best prognostic predictive power^13^, which is similar to our MSE results. DFA as a scaling analysis method to determine the statistical self-affinity of a signal can be used for the evaluation of the fractal behavior in the heart beat dynamics. The short-term (α1; 4–11 beats,) and long-term (α2; 11–64 beats) fractal correlation exponents have been shown to provide a clearer understanding of the fractal correlation property in a physiological system^45^. DFA has also been associated with the interaction between sympathetic and vagal systems^46^. In the DIAMOND-CHF trial, after adjusting for clinical parameters, DFAα1 but not linear parameters remained to be an independent predictor of mortality^14^. Taken together with our findings, non-linear HRV analysis may be a useful tool to evaluate the risk of cardiovascular events.

The traditional linear HRV parameters have also been positively associated with CVD risk factors and multiple cardiovascular outcomes including coronary artery disease and cardiovascular mortality^47,48^. We also found similar results in that linear HRV parameters including SDRR, VLF, LF, HF and LF/HF ratio were also significantly associated with AAC. In addition, combining linear and non-linear analysis further significantly improved the discriminatory power of the severity of AAC. Combining these linear and non-linear HRV parameters can provide more accurate information to build a ROC curve model to predict the severity of AAC.

There are several limitations to this study. First, this is a small pilot study and the findings should be confirmed by a larger clinical study with long-term follow-up data. Second, our study group is limited to PD patients, and further studies are needed to elucidate whether the same association between AAC and heart rhythm complexity exists in hemodialysis patients.

In conclusion, heart rhythm complexity analysis can predict the severity of AAC in PD patients. DFAα1 had the greatest discriminatory power to differentiate PD patients with higher or lower AAC. In addition, DFAα1 and MSE slope 1–5 significantly improved the discriminatory power of the linear parameters, which suggests the advantage of combining linear and non-linear parameters.

## Methods

### Patients

We prospectively enrolled 133 patients who received PD with conventional glucose-based lactate-buffered solution (UltraBag; Baxter Healthcare SA, Singapore) for more than 6 months. Patients with chronic atrial fibrillation, clinical signs of acute infection, and those with a prior renal transplant were excluded. The baseline characteristics, medical history and medication usage were carefully recorded, and biochemical parameters were measured during initial evaluation. All patients received 24-h ambulatory ECG Holter recording (ZymedDigiTrak Plus 24-Hour Holter Monitor Recorder and Digitrak XT Holter Recorder 24 Hour, Philips, Amsterdam, Netherlands). This study was approved by the Institutional Review Board of National Taiwan University Hospital, and all research was performed in accordance with relevant guidelines and regulations. All subjects provided written informed consent including for storage of their information in the hospital database and usage for research.

### Data pre-processing

A stable 4 hours segment of daytime RR intervals (between 9AM and 5PM) was selected for analysis. The selected electrocardiograms were automatically annotated via an algorithm and carefully examined by two experienced technicians.

### Time and frequency domain analysis

All parameters were calculated according to the recommendations of the North American Society of Pacing Electrophysiology and the European Society of Cardiology^10^. SDRR and the percentage of absolute differences in normal RR intervals greater than 50 ms (pNN50) were calculated to represent the total variance and vagal modulation of heart rate. The frequency domain parameters including high frequency (HF; 0.15–0.4 Hz), low frequency (LF; 0.04–0.15 Hz), and very low frequency (VLF; 0.003–0.04 Hz) power, were computed by averaging the absolute powers (ms^2^) after Fourier transformation.

### Detrended fluctuation analysis (DFA)

DFA is used to evaluate the fractal behavior beneath seemingly nonstationary RR dynamics by eliminating extrinsic trends to remove spurious long-term correlations. The external trends were assumed to be the linear or polynomial fitted trends over different scales, and by removing these trends from the integrated time series, the intrinsic fractal behavior could be better quantified. Detrended fluctuations were calculated by adding up the detrended integrated time series in individual scales. Then, the logarithmic plot of fluctuations against time scales were further constructed. The slope (α exponent) of the log-log plot was used to indicate the fractal correlation characters of time series.

While the respiratory sinus arrhythmia is responsible for most of the short-term RR dynamics in normal subjects, the crossover phenomenon of α exponents of RR dynamics over short (α1; 4–11 beats) and long (α2; 11–64 beats) time scales are of importance. We calculated both short- and long-term α exponents for better probing the fractal characters of the biological system.

### Multiscale entropy (MSE) analysis

MSE takes the predictability of multiple time scales into account and extends the entropy of a single timescale to the information richness structure embedded over different time scales. The profile of the sequential changes of the entropies over different time scales can be further quantifies. In brief, the time series of different time scales were derived by using a coarse-graining process (i.e. averaging consecutive beats to form a new time series), and the sample entropy was adopted to estimate the predictability over different time scales^49^. The estimated entropy over different time scales can then be used to represent the complexity (meaningful information richness) of the physiological signals. The linear-fitted slope of scale 1 to scale 5 (slope 1–5), the sum of entropy values of scales 1 to scale 5 (area 1–5) or scale 6 to scale 20 (area 6–20) were calculated to quantify the complexity of the beat-to-beat dynamics exhibited in short and long time scales.

### Echocardiography

Transthoracic echocardiography (iE33 xMATRIX Echocardiography System, Philips, Amsterdam, Netherlands) was performed in all patients. The LVEF was quantitated by M-mode measurements or area-length methods^9^.

### Computed tomography

A standard 64-MDCT scan (LightSpeed VCT, GE Healthcare, Milwaukee, WI) was performed in all patients. The calcified area was quantified based on an attenuation range of >150 Hounsfield units using image analysis software (ImageJ, version 1.45, National Institutes of Health, Bethesda, MD). The percentages of the area of the whole aorta affected by aortic calcification were calculated from the images of four consecutive slices just above the iliac bifurcation level^9,32^.

### Statistical analysis

Data were expressed as median (25^th^ and 75^th^ percentiles). Comparisons of data between the higher and lower AAC groups were performed by the Mann-Whitney U test. Differences between proportions were calculated by the chi-square test or Fisher’s exact test. Logistic regression analysis was used to validate associations between parameters and the presence of high AAC. Significant determinants in univariate logistic regression analysis (P < 0.05) were then tested in multivariate logistic regression analysis with stepwise subset selection to identify independent factors to predict the presence of high AAC. Linear regression analysis was used to validate associations between parameters and percentage of AAC. Significant determinants in univariate linear regression analysis (P < 0.05) were then tested in multivariate linear regression analysis with stepwise subset selection to identify independent factors to predict the percentage of AAC. The goodness-of-fit of a logistic model was indicated by R^2^, while the discriminatory power of the model was assessed by the area under the ROC curve (AUC).

Two statistics, net reclassification improvement (NRI) and integrated discrimination improvement (IDI), were used to evaluate improvements in the accuracy of the prediction after adding a single nonlinear parameter into a logistic regression model using only linear parameters^50^. The significance of NRI and IDI statistics was based on approximate normal distributions. All statistical analyses were performed by R software (http://www.r-project.org/) and SPSS version 25 for Windows (SPSS Inc., IL, USA). The significance level of the statistical analysis was set at 0.05.
